# A Simple Mechanism for Beyond-Pairwise Correlations in Integrate-and-Fire Neurons

**DOI:** 10.1186/s13408-015-0030-9

**Published:** 2015-09-01

**Authors:** David A. Leen, Eric Shea-Brown

**Affiliations:** Department of Applied Mathematics, University of Washington, Seattle, WA USA; Department of Physiology and Biophysics, University of Washington, Seattle, WA USA; Program in Neuroscience, University of Washington, Seattle, WA USA; Allen Institute for Brain Science, Seattle, WA USA

**Keywords:** Spike correlations, Higher-order correlations, Maximum entropy model, Common input, Dichotomous Gaussian model, Generalized linear model, Linear-nonlinear cascade, Population code

## Abstract

The collective dynamics of neural populations are often characterized in terms of *correlations* in the spike activity of different neurons. We have developed an understanding of the circuit mechanisms that lead to correlations among cell pairs, but little is known about what determines the population firing statistics among larger groups of cells. Here, we examine this question for a simple, but ubiquitous, circuit feature: common fluctuating input arriving to spiking neurons of integrate-and-fire type. We show that this leads to strong beyond-pairwise correlations—that is, correlations that cannot be captured by maximum entropy models that extrapolate from pairwise statistics—as for earlier work with discrete threshold crossing (dichotomous Gaussian) models. Moreover, we find that the same is true for another widely used, doubly stochastic model of neural spiking, the linear–nonlinear cascade. We demonstrate the strong connection between the collective dynamics produced by integrate-and-fire and dichotomous Gaussian models, and show that the latter is a surprisingly accurate model of the former. Our conclusion is that beyond-pairwise correlations can be both broadly expected and possible to describe by simplified (and tractable) statistical models.

## Introduction

Interest in the collective dynamics of neural populations is rapidly increasing, as new recording technologies yield views into neural activity on larger and larger scales, and new statistical analyses yield potential consequences for the neural code [4, 5, 9, 20, 28, 34]. A fundamental question that arises as we seek to quantify these population dynamics is the statistical *order* of correlations among spiking activity in different neurons. That is, can the co-dependence of spike events in a set of neurons be described by an (overlapping) set of correlations among pairs of neurons, or are there irreducible higher-order dependencies as well? Recent studies show that purely pairwise statistical models are successful in capturing the spike outputs of neural populations under some stimulus conditions [22, 27, 28]. At the same time, different stimuli or different (or larger) populations can produce beyond-pairwise correlations [9, 16, 18, 31, 33]. In these studies, and in the present paper, beyond-pairwise correlations are defined by comparing with a pairwise maximum entropy (PME) model of spike trains: that is, a statistical model built with minimal assumptions about collective spiking beyond the rates of spiking in single cells and correlations in the spikes from cell pairs.

Despite these rich empirical findings, we are only beginning to understand what *dynamical* features of neural circuits determine whether or not they will produce substantial beyond-pairwise *statistical* correlations. Recent work has suggested that one of these mechanisms is common—or correlated—input fluctuations arriving simultaneously at multiple neurons [1, 2, 10, 14, 23]; importantly, this is a feature that occurs in many neural circuits found in biology [3, 24, 32]. In particular, [1, 14] showed that common, Gaussian input fluctuations, when “dichotomized” so that inputs over a given threshold produce spikes, give rise to strong beyond-pairwise correlations in the spike output of large populations of cells. This is an interesting result, as a step function thresholding mechanism produces beyond-pairwise correlations in spike outputs starting with purely pairwise (Gaussian) inputs.

A natural question is whether more realistic, dynamical mechanisms of spike generation—beyond “static” step function transformations—will also serve to produce strong beyond-pairwise correlations based on common input processes. In this paper, we show that the answer is yes, and connect several widely used models of neural spiking to explain why. In particular we show that, in contrast to the PME, the dichotomous Gaussian model gives a highly accurate description of the complete correlation structure of an integrate-and-fire population with common inputs.

## Results

### An Exponential Integrate-and-Fire Population with Common Inputs

**Fig. 1:**
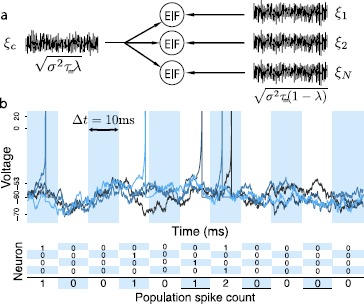
(**a**) A population of $N=3$ EIF neurons receiving common $\xi_{c}$ and independent inputs $\xi_{i}$. The voltages of the neurons evolve according to Eq. (1). Parameters: $\tau _{m} = 5\mbox{ ms}$, $\varDelta_{T} = 3\mbox{ mV}$, $V_{T} = 20\mbox{ mV}$, $V_{S} = -53\mbox{ mV}$, $V_{R} = -60\mbox{ mV}$, $\tau_{\mathrm{ref}} = 3\mbox{ ms}$. We tune the noise amplitude so that when the DC component of the input is $\gamma= -60\mbox{ mV}$, the neurons fire at 10 Hz; this yields $\sigma= 6.23\mbox{ mV}$. The resulting firing is strongly irregular, with the coefficient of variation of the ISI distribution being 0.91. (**b**) Cartoon of the binning process: spikes recorded from each of the EIF neurons in a bin contribute towards the population spike count. More than one spike occurring *from the same neuron* within a single bin is treated as a single event. This happens less than 0.4 % of the time in our numerical simulations with $\mu=0.1$ and $\rho=0.1$ (input parameters $\gamma=-60\mbox{ mV}$, $\sigma =6.23\mbox{ mV}$, $\lambda=0.30$)

Figure 1 shows a ubiquitous situation in neural circuitry: a group of cells receiving fluctuating common input. We model this in a homogeneous population of *N* exponential integrate-and-fire (EIF) neurons [6, 8]. Each cell’s membrane voltage $V_{i}$, $i=1,\ldots,N$, evolves according to

1 $$ \begin{aligned} \tau_{m} V_{i}^{\prime}&= -V_{i} +\psi(V_{i})+I_{i}(t), \\ I_{i}(t) &= \gamma+\sqrt{\sigma^{2}\tau_{m}} \bigl[\sqrt{1-\lambda}\xi _{i}(t)+\sqrt{\lambda}\xi_{c}(t) \bigr] , \end{aligned} $$

where $\psi(V_{i}) =\varDelta_{T} \exp{ ((V_{i} - V_{S})/\varDelta_{T} )}$. Here, $\tau_{m}=5\mbox{ ms}$ is the membrane time constant, $\varDelta_{T}= 3\mbox{ mV}$ gives the slope of the spike initiation, and $V_{S}= -53\mbox{ mV}$ is the “soft” threshold for spike initiation. When voltages cross $V_{S}$, they begin to diverge rapidly; when they later cross a “hard” threshold $V_{T} = 20\mbox{ mV}$, they are said to fire a spike and are reset to the value $V_{R} = -60\mbox{ mV}$. Voltages are then held at that voltage for a refractory period $\tau_{\mathrm{ref}} = 3\mbox{ ms}$. See the caption of Fig. 1 for further parameter values, which drive the cell to fire with the typically observed irregular, Poisson-like statistics [29].

Each cell’s input current $I_{i}(t)$ has a constant (DC) level *γ*, and a white noise term with amplitude *σ*. The noise term has two components. The first is the common input $\xi_{c}(t)$, which is shared among all neurons. The second is an independent white noise $\xi_{i}(t)$; the relative amplitudes are scaled so that the inputs to different cells are correlated with (Pearson’s) correlation coefficient *λ* (as in, e.g., [7, 13, 26], cf. [3]).

We quantify the population output by binning spikes with temporal resolution $\Delta t = 10\mbox{ ms}$ (see Fig. 1). (On rare occasions (<0.4 % of the bins; see Fig. 1, caption) multiple spikes from the same neuron can occur in the same bin. These are considered as a single spike.) The spike *firing rate* is quantified by *μ*, the probability of a spike occurring in a bin for a given neuron. *Pairwise correlation* in the simultaneous spiking of neurons *i*, *j* is quantified by the correlation coefficient $\rho =\operatorname{Cov}(n_{i},n_{j})/\mathrm{Var}$, where $n_{i}$, $n_{j}$ are the $\{0,1\}$ spike events for the cells and Var is their (identical) variance $\mu (1-\mu)$.

### Emergence of Strong Beyond-Pairwise Correlations in EIF Populations

**Fig. 2:**
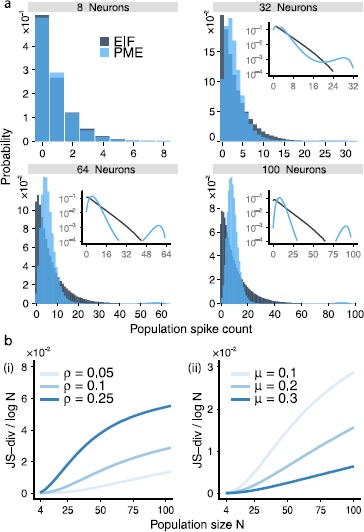
(**a**) Population spike-count distributions $P_{\mathrm{EIF}}(k)$ for the EIF and $P_{\mathrm{PME}}(k)$ for the pairwise maximum entropy (PME) model, for populations of $N= 8, 32, 64$, and 100 neurons. Here $\mu= 0.1$ and $\rho= 0.1$ (input parameters $\gamma=-60\mbox{ mV}$, $\sigma=6.23\mbox{ mV}$, $\lambda=0.30$). The distributions $P_{\mathrm{EIF}}(k)$ and $P_{\mathrm{PME}}(k)$ are similar for smaller populations but differ larger populations. *Inset*: the same distributions on a log-linear scale. (**b**) The Jensen–Shannon (JS) divergence between the EIF and the pairwise maximum entropy (PME) model. We normalize by $\log(N)$, the natural growth rate of the JS divergence. *Left*: JS divergence for a constant value of $\mu= 0.1$ and increasing values of correlation *ρ* (input parameters $\gamma =-60\mbox{ mv}$, $\sigma=6.23\mbox{ mV}$, $\lambda=0.17$, 0.30, and 0.59, respectively). *Right*: JS divergence for constant value of $\rho= 0.1$ and increasing values of firing rate *μ* vs. population size. The firing rate was varied by increasing the DC component of the input current, *γ* (input parameters $\sigma=6.23\mbox{ mV}$, $\gamma=-60\mbox{ mV}$, −58.2 mV, and −56.8 mV, respectively, and $\lambda=0.30$, 0.25, and 0.23, respectively). The JS divergence grows with increasing *ρ* and decreasing *μ*

Beyond these statistics of single cells and cell pairs, we describe multineuron activity via the distribution of population spike counts—i.e., the probability $P_{\mathrm{EIF}}(k)$ that *k* out of the *N* cells fire simultaneously (as in, e.g., [1, 2, 14, 16]). Figure 2(a) illustrates these distributions. The question we ask is: Do beyond-pairwise correlations play an important role in determining the population-wide spike-count distribution?

To answer this, we compare the population spike-count distribution $P_{\mathrm{EIF}}(k)$ from the EIF model against that which would be predicted for a pairwise maximum entropy (PME) model of spiking neurons. The PME model matches the spike probability *μ* for each neuron and pairwise spike correlation *ρ* for each pair of neurons, while making minimal further assumptions on the joint probability distribution [9, 16, 18, 31, 33], cf. [15, 30]. For a population of *N* neurons with identical means *μ* and pairwise correlations *ρ*, as for our simple circuit model, the PME model gives a distribution of population spike counts,

$$P_{\mathrm{PME}}(k)= Z^{-1} \binom{N}{k}\exp{\bigl(\alpha k + \beta k^{2}\bigr)} , $$

where *Z* is a normalization factor and parameters *α* and *β* are adjusted numerically [14]. (Specifically, we use the function fminunc to find parameters *α* and *β* which minimize the negative likelihood of spike counts *k* from simulations of the EIF model, under the model $P_{\mathrm{PME}}(k)$.)

Figure 2(a) demonstrates that, for small populations, the corresponding PME and EIF distributions are similar. However, for populations larger than about $N=30$ neurons, strong differences emerge. This difference in population spike-count distributions demonstrates that the EIF model produces beyond-pairwise correlations that strongly impact the structure of population firing. This is because the moments of the population spike-count distribution at a given order are determined by the moments—and hence correlations—of spikes among sets of cells of up to that order. Because the PME and EIF models have matched first- and second-order moments but different population spike-count distributions, they must differ in their beyond-pairwise correlations.

We quantify the discrepancy between $P_{\mathrm{PME}}(k)$ and $P_{\mathrm{EIF}}(k)$ via the (normalized) Jenson–Shannon (JS)-divergence $\mbox{JS-div}/ \log(N)$,

2 $$ \mbox{JS-div} = \frac{1}{2} D \bigl( P_{\mathrm{PME}}(k) \| M(k) \bigr) + \frac{1}{2}D \bigl( P_{\mathrm{EIF}}(k) \| M(k) \bigr), $$

where the “averaged” distribution $M(k)=\frac{1}{2} P_{\mathrm{PME}}(k) + \frac{1}{2} P_{\mathrm{EIF}}(k)$ and $D ( \cdot\| \cdot )$ is the Kullback–Leibler divergence [12]. See Fig. 2(b), which shows very similar results for the EIF system to those found for a thresholding model in [14] (see below). In particular, the EIF model produces departures from the PME model for a wide range of correlations *ρ* and mean firing rates *μ*. Additionally, as in [14] (cf. [17]), the Jensen–Shannon divergence grows with increasing population size *N*. Moreover, the divergence increases with increasing pairwise correlation and decreasing mean firing rate.

### A Linear–Nonlinear Cascade Model That Approximates EIF Spike Activity and Produces Beyond-Pairwise Correlations

We next study the impact of common input on beyond-pairwise correlations in a widely used point process model of neural spiking. This is the linear–nonlinear cascade model, where each neuron fires as a (doubly stochastic) inhomogeneous Poisson process. We use a specific linear–nonlinear cascade model that is fit to EIF dynamics. This both establishes that the common input mechanism is sufficient to drive beyond-pairwise correlations in the cascade model, and develops a semi-analytic theory for the population statistics in the EIF system.

In the linear–nonlinear cascade, each neuron fires as an inhomogeneous Poisson process with rate given by convolving a temporal filter $A(t)$ with an input signal $c(t)$ and then applying a time independent nonlinear function *F* [19]:

$$r(t) = F \bigl(A * c(t) \bigr) . $$

**Fig. 3:**
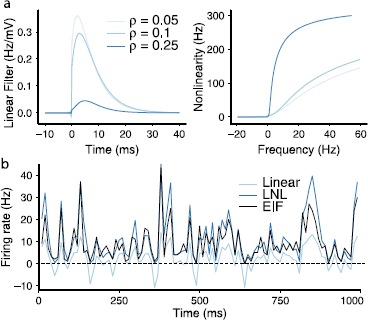
(**a**) The linear filter $A(t)$ and static nonlinearity *F* computed for inputs that yield several values of the spike correlation coefficient *ρ*. The filter receives a noise amplitude of $\sigma\sqrt{1-\lambda}$. The static nonlinearity receives a noise amplitude of *σ*. (**b**) The static nonlinearity applied to the linear estimate of the firing rate, for $\mu= 0.1$, $\rho= 0.1$, plotted over a randomly chosen 1000 ms time interval. The nonlinearity increases the firing rate magnitude and rectifies negative firing rates. This gives the predicted firing rates shown in *blue*; comparing with firing rates computed by binning spikes in 10 ms windows from simulations of the EIF model, shown in *black*, shows that the LNL is a fairly accurate model of the EIF dynamics

The signal for each cell is the common input $c(t) = \sqrt{\sigma^{2} \tau\lambda} \xi_{c}(t)$. The filter $A(t)$ is computed as the linear response of the firing rate to a weak input signal, via an expansion of the Fokker Planck equation for Eq. (1) around the equilibrium obtained with “background” current $\gamma+ \sqrt {\sigma ^{2} \tau(1-\lambda)} \xi(t)$. This calculation follows exactly the methods described in [21]. For the static nonlinearity, we follow [19] and take

$$F(x) = \varPhi \biggl( \gamma+ \frac{x}{\varPhi^{\prime}(\gamma)} \biggr) , $$

where $\varPhi ( \gamma )$ is the equilibrium firing rate obtained at the background currents described above. This choice, in particular, ensures that we recover the linear approximation $r(t) = A * c(t)$ for weak input signals. For EIF neurons, the linear filter must be approximated numerically, hence the semi-analytic nature of our model. The numerical approximations for the filter, nonlinearity, and resulting firing rate are shown in Fig. 3.

For an inhomogeneous Poisson process with rate $r(t)$ conditioned on a common input $c(t)$, the probability of at least one spike occurring in the interval $[t,t+\Delta t]$ is

3 $$\begin{aligned} P(\mathrm{spike}\in\Delta t | c ) =& 1 - \exp{ \biggl(-\int_{t}^{t + \Delta t} r(s) \,\mathrm {d}s \biggr)} \end{aligned}$$

4 $$\begin{aligned} =& 1 - \exp(-\mathcal{S}) \equiv\tilde{L}(\mathcal{S}), \end{aligned}$$

where we have defined $\mathcal{S} = \int_{t}^{t + \Delta t} r(s) \,\mathrm {d}s $.

Conditioned on the common input—or, equivalently, the windowed firing rate $\mathcal{S}$—each of the *N* neurons produces spikes independently. Thus, the probability of *k* cells firing simultaneously is

5 $$ P_{\mathrm{LNL}}(k) = \binom{N}{k} \int_{-\infty}^{ \infty} \phi _{\mathrm{LNL}}(\mathcal{S}) \bigl(1-\tilde{L}(\mathcal{S}) \bigr)^{N-k} \tilde {L}(\mathcal{S})^{k} \,\mathrm {d}\mathcal{S}, $$

where $\phi_{\mathrm{LNL}}(\mathcal{S})$ is the probability density function for $\mathcal{S}$, which we estimate numerically via the linear filter *A* and static nonlinearity *F* described above. We note that [25] derive a related expression for a different definition of synchronous output for a neural population.

**Fig. 4:**
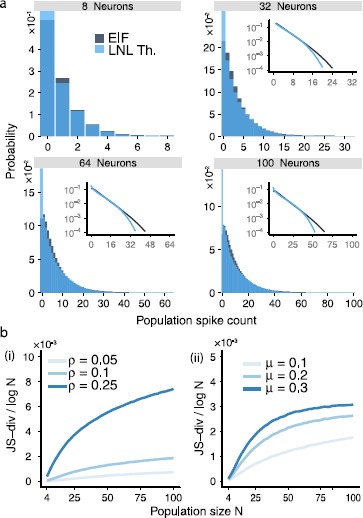
(**a**) Population spike-count distributions $P_{\mathrm{EIF}}(k)$ for the EIF model and $P_{\mathrm{LNL}}(k)$ for the linear–nonlinear cascade approximation for 8, 32, 64, and 100 neurons for $\mu= 0.1$ and $\rho= 0.1$. While the distributions are very similar overall, the LNL model greatly overestimates the zero population spike-count probabilities and underestimates the tails. *Inset*: the same distributions on a log-linear scale. (**b**) The JS divergence between the EIF and LNL is an order of magnitude smaller than PME. (Also, the order of the mean firing rates is reversed when compared to the PME as the LNL cascade gives a better approximation at lower firing rates)

Figure 4(a) shows that the LNL cascade captures the general structure of the EIF population output across a range of population sizes. In particular, it produces an order-of-magnitude improvement over the PME model—see JS-divergence values in Fig. 4(b)—and reproduces the skewed structure produced by beyond-pairwise correlations.

This said, the LNL model does not produce a perfect fit to the EIF outputs, the most obvious problem being the overestimation of the zero spike probabilities, which in the $N=100$ case are overestimated by almost 100 % (the tail probabilities are also underestimated). Notably, the LNL fits become almost perfect for lower correlations i.e. $\rho= 0.05$ (data not shown). This suggests the discrepancies are due to failures of the LNL approximation for large fluctuations in the instantaneous spiking rates $r(t)$ (see Fig. 3(b)); these fluctuations are smaller at lower correlation values, which lead to smaller signal currents in the LNL formulation. While further work would be required to trace the precise origin of this discrepancy, we conjecture that one factor is the lack of a refractory period in the LNL model, which will impact firing statistics most strongly during and after fluctuations to high instantaneous rates.

### The Dichotomized Gaussian (DG) Model Gives an Excellent Description of the EIF Population Activity

So far we have studied the emergence of beyond-pairwise correlations in two spiking neuron models—the EIF model, described in terms of a stochastic differential equation, and the LNL model, which is a continuous-time reduction of the EIF to a doubly stochastic point process. Next, we show how these results connect to earlier findings for a more general and abstracted statistical model. This is the Dichotomous Gaussian (DG) model, which has been shown analytically to produce beyond-pairwise correlations and to describe empirical data from neural populations [1, 2, 14, 33].

In the DG framework, spikes either occur or fail to occur independently and discretely in each time bin. Specifically, at each time *N* neurons receive a correlated Gaussian input variable with mean *γ* and correlation *λ*. Each neuron applies a step nonlinearity (Heaviside function) to its inputs, spiking only if its input is positive. Input parameters *γ* and *λ* are chosen to match two target firing statistics: the spike rate *μ* and the correlation coefficient *ρ*.

**Fig. 5:**
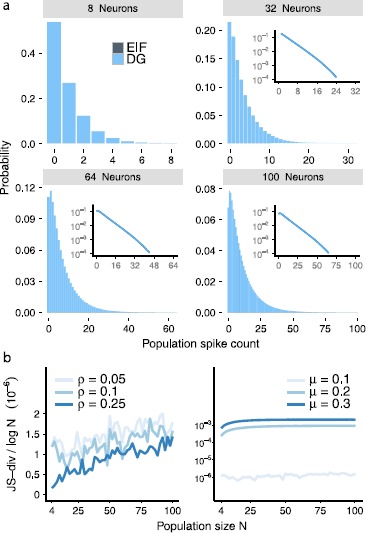
(**a**) Population spike-count distributions $P_{\mathrm{EIF}}(k)$ for the EIF model and $P_{\mathrm{DG}}(k)$ for the Dichotomous Gaussian (DG) approximation for 8, 32, 64, and 100 neurons for $\mu= 0.1$ and $\rho= 0.1$. The distributions are very similar, showing that the DG model very accurately captures EIF spiking statistics. Compare with Figs. 2, 4. *Inset*: the same distributions on a log-linear scale. (**b**) *Left*: JS divergence between the EIF and DG models for a constant value of $\mu= 0.1$ and increasing values of correlation *ρ*; values appear noisy, but are several orders of magnitude lower than the JS divergence between EIF and PME or LNL models in Figs. 2(b), 4(b). *Right*: Similar, for a constant value of $\rho= 0.1$ and increasing values of firing rate *μ*. The JS divergence grows with increasing *μ*

In Fig. 5, we compare the population output of the DG model with that from the EIF model. We see that, once the two models are constrained to have the same pairwise correlation *ρ* and firing rate *μ*, the rest of their population statistics match almost exactly over the full range of population sizes, for firing rates $\mu =0.1$ and a variety of correlation values *ρ*. Panel b(ii) shows that the match degrades somewhat for higher firing rates.

**Fig. 6:**
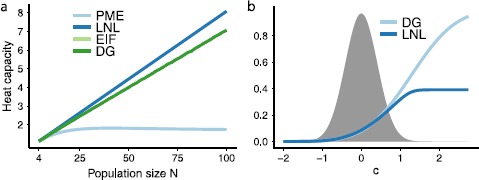
(**a**) The heat capacity increases linearly for the LNL cascade, the EIF and the DG models. The heat capacity of the LNL cascade increases at a slightly greater rate than that of the EIF and DG models, which overlap. The heat capacity for the PME model saturates at a population of approximately $N=30$ neurons. (**b**) Comparing the $L(c)$ vs. the $\tilde{L}( f(c))$ functions for the DG and LNL models, respectively (here input correlations $\lambda=0.17$ for DG model). The two functions largely agree over about 2 standard deviations of the Gaussian pdf $\phi_{\mathrm{DG}}(c)$ (*shaded*)

Figure 6(a) provides another view into the similar population statistics produced by the different models. Here, we study the “heat capacity” $C = \operatorname{Var} (\log_{2} P (k) ) / N$, which is a measure of how variable the probabilities of different population spike counts *k* are. In prior work [14] it was shown that this statistic grows linearly (i.e., extensively) with population size *N* for the DG model, and the figure shows that the same holds for the EIF and LIF models. This growth stands, as first noted by [14], in marked contrast to the heat capacity for the PME model, which saturates at a population of approximately $N = 30$ neurons.

We next develop the mathematical connection between the DG and the EIF models, via our description of the LNL model above.

First, we note that, for the DG model, the correlated Gaussian input that each neuron receives can be written, for the *i*th neuron, $Z_{i} = \gamma+ \sqrt{1-\lambda} T_{i} + \sqrt{\lambda} c$. Here, $T_{i}$ is a Gaussian random variable (with unit variance) which is independent for each neuron (the independent input), *c* is a Gaussian random variable that is common input to all neurons (the common input), and *γ* is a constant term giving the mean input. The probability of a spike is given by a step function applied to the input. For a given realization of the common input *c*, this is $P(Z_{i} > 0 | c)$. We can again define a “*L*” function similar to that in Eq. (4):

6 $$ L(c) = P \biggl( T_{i} > \frac{- \sqrt{\lambda}c-\gamma}{\sqrt {1-\lambda}} \biggr) = \operatorname{CDF} \biggl(\frac{\sqrt{\lambda}c+\gamma}{\sqrt {1-\lambda }} \biggr) . $$

Here, the CDF is the cumulative distribution function for a Gaussian variable with unit variance (and the equality follows from the symmetry of this distribution). Equipped with Eq. (6), the probability of observing a spike count *k* is similar to Eq. (5):

7 $$ P_{\mathrm{DG}}(k) = \binom{N}{k} \int_{-\infty}^{ \infty} \phi_{\mathrm{DG}}(c) \bigl(1-{L}(c) \bigr)^{N-k} {L}(c)^{k} \,\mathrm {d}c, $$

where $\phi_{\mathrm{DG}}(c)$ is the pdf of a one-dimensional Gaussian with mean 0 and variance *λ*.

We next compare the population spike-count distributions $P_{\mathrm{LNL}}(k)$ and $P_{\mathrm{DG}}(k)$. To make the comparison we must transform from the probability density function of the linear–nonlinear model $\phi_{\mathrm{LNL}}$ to the Gaussian pdf $\phi_{\mathrm{DG}}$ using the nonlinear change of variable:

8 $$ \mathcal{S} = f(c), \quad\text{where } f^{\prime}(c) = \frac{\phi _{\mathrm{DG}}(c)}{\phi_{\mathrm{LNL}} (f(c) )}. $$

Writing the LNL-cascade probability in terms of the *c* variable we obtain

9 $$ P_{\mathrm{LNL}}(k) = \binom{N}{k}\int_{-\infty}^{ \infty} \phi _{\mathrm{DG}}(c) \bigl(1-\tilde{L}\bigl(f(c)\bigr) \bigr)^{N-k} \tilde{L}\bigl(f(c)\bigr)^{k} \,\mathrm {d}c. $$

Thus, after the transformation the only difference between the LNL and DG models is the functions $L(c)$ vs. $\tilde{L}( f(c))$. Figure 6(b) shows that these functions largely agree over about 2 standard deviations of the Gaussian pdf of values of the common input signal *c*.^1^ This reveals why the LNL and DG—and, by extension, the EIF—models all produce such similar population-level outputs, including their higher-order structure.

## Conclusion

We have shown that Exponential-Integrate-and-Fire (EIF) neurons receiving common input give rise to strong beyond-pairwise correlations—that is, distributions of population spike counts that cannot be described by a pairwise maximum entropy (PME) approach. Moreover, the population output that results can be predicted from a linear–nonlinear (LNL) cascade model, which forms a tractable reduction of the EIF neuron. Beyond giving an explicit formula for the EIF population spike-count distribution, our findings for the LNL-cascade model demonstrate that common input will drive beyond-pairwise correlations in a widely used class of point process models.

Finally, we show that there is a surprisingly exact connection between the population dynamics of the EIF- and LNL-cascade models and that of the (apparently) simpler Dichotomized Gaussian (DG) model of [1, 14]. The success of the DG model in capturing EIF population statistics is significant for two reasons. First, it suggests one reason why this abstracted model has been able to capture the population output recorded from spiking neurons [33]. Second, because the DG model is a special case of a Bernoulli generalized linear model (see the appendix), our finding indicates that this very broad class of statistical models may be able to capture the higher-order population activity in neural data. A key feature of these models would be the inclusion of common fluctuations in the spike probabilities of cells in each time bin (cf. [11]); such models can also be extended to include spike history-dependent terms. This would then capture an effect missing here: temporal correlations in spike trains (e.g., refractory effects).

## Appendix: Generalized Linear models

The LNL model provides a reduction of the EIF model to an inhomogeneous Poisson process that is based directly on the underlying SDEs, and is in extremely wide use in neural modeling [6]. However, it is far from the only approach to statistical modeling of spiking neurons. In particular, generalized linear models can be fit to the Bernoulli data given by the 1’s and 0’s of binned spikes in individual cells. Such models similarly apply a linear filter to the common input signal, and followed by a static nonlinearity $f(\cdot)$, to yield a spiking probability for the current time bin. Noting that any linear filter on our (Gaussian white noise) input signal will yield a Gaussian value *s*, this class of models therefore yields spiking probabilities $f(s)$ where *s* is Gaussian. Comparing with Eq. (6) in the main text, we see that the DG and generalized linear models have the same general form, when *f* is taken to be the cumulative distribution function for a Gaussian (as in “probit” models).

## Footnotes

1 At large values of common input *c*, the higher values of $L(c)$ for the DG model account for its closer correspondence to the EIF model in the tails of $P(k)$ in Fig. 5.
